# Music structure determines heart rate variability of singers

**DOI:** 10.3389/fpsyg.2013.00334

**Published:** 2013-07-09

**Authors:** Björn Vickhoff, Helge Malmgren, Rickard Åström, Gunnar Nyberg, Seth-Reino Ekström, Mathias Engwall, Johan Snygg, Michael Nilsson, Rebecka Jörnsten

**Affiliations:** ^1^Center for Brain Repair and Rehabilitation, Institute of Neuroscience and Physiology, Sahlgrenska Academy, University of GothenburgGothenburg, Sweden; ^2^Department of Philosophy, Linguistics and Theory of Science, University of GothenburgGothenburg, Sweden; ^3^Professional Musician and ComposerMusikalliansen, Torslanda, Sweden; ^4^Department of Clinical Physiology, Sahlgrenska University HospitalGothenburg, Sweden; ^5^Cantor, The Swedish Church, Sätila ParishHällingsjö, Sweden; ^6^Department of Cultural Sciences, University of GothenburgGothenburg, Sweden.; ^7^Department of Anaesthesia and Intensive Care, Sahlgrenska University HospitalGothenburg, Sweden; ^8^Hunter Medical Research Institute, University of NewcastleNewcastle, NSW, Australia; ^9^Mathematical Sciences, University of Gothenburg and Chalmers University of TechnologyGothenburg, Sweden

**Keywords:** choral singing, heart rate variability, respiratory sinus arrhythmia, frequency analysis, autonomic nervous system

## Abstract

Choir singing is known to promote wellbeing. One reason for this may be that singing demands a slower than normal respiration, which may in turn affect heart activity. Coupling of heart rate variability (HRV) to respiration is called Respiratory sinus arrhythmia (RSA). This coupling has a subjective as well as a biologically soothing effect, and it is beneficial for cardiovascular function. RSA is seen to be more marked during slow-paced breathing and at lower respiration rates (0.1 Hz and below). In this study, we investigate how singing, which is a form of guided breathing, affects HRV and RSA. The study comprises a group of healthy 18 year olds of mixed gender. The subjects are asked to; (1) hum a single tone and breathe whenever they need to; (2) sing a hymn with free, unguided breathing; and (3) sing a slow mantra and breathe solely between phrases. Heart rate (HR) is measured continuously during the study. The study design makes it possible to compare above three levels of song structure. In a separate case study, we examine five individuals performing singing tasks (1–3). We collect data with more advanced equipment, simultaneously recording HR, respiration, skin conductance and finger temperature. We show how song structure, respiration and HR are connected. Unison singing of regular song structures makes the hearts of the singers accelerate and decelerate simultaneously. Implications concerning the effect on wellbeing and health are discussed as well as the question how this inner entrainment may affect perception and behavior.

## Introduction

This paper aims to illuminate and discuss how singing (Grape et al., 2003) and especially choir singing (Marti, 2012) promotes wellbeing. We focus on the interplay between two oscillators: the respiratory organ and the heart, while singing. The heart does not keep a constant rhythm. On the contrary, heart rate (HR) is accelerating and decelerating constantly. This fluctuation in HR is called heart rate variability (HRV). In this article, we mainly speak of the dynamic aspects of HRV, i.e., how HR develops over time forming cycles of HR fluctuations, but we also measure HRV by RMSSD (Root Mean Square of the Successive Differences) (Degiorgio et al., 2010).

It is known that HRV and respiration rate affect each other. This interdependency, first discussed by (Ludwig, 1847), is referred to as respiratory sinus arrhythmia (RSA). The heart and the respiratory system are irregular oscillators and the interaction is weak (Schafer et al., 1998). If, however, respiration is guided, one of the oscillators becomes regular. If this pace is slow the synchronization dependence between the two oscillators increases and becomes stronger: slow respiration produces higher HRV amplitudes (Song and Lehrer, 2003).

The primary pacemaker of the heart is the sinoatrial node located in the right atrium. The node is populated by millions of cells, each a small oscillator with a natural frequency of its own (Ostborn et al., 2003). In the healthy heart these frequencies are phase locked, enabling the node to produce regular contraction commands. The sinoatrial node has a natural frequency of about 90 beats per min (Sukhova and Mazurov, 2007). The node is affected by the autonomous nervous system (ANS). The sympathetic nervous system and the vagal nerve transmit impulses from a cardiopulmonary oscillator consisting of interneurons connecting brainstem nuclei (the nucleus of the solitary tract and the nucleus ambiguus) (Richter and Spyer, 1990; Porges, 1995). In addition, pulmonary stretch receptors are involved in the neural mediation of RSA (Taha et al., 1995). Furthermore, exhalation affects baroreflex sensibility causing a vagal response which slows down the heart (Sleight et al., 1995; Bernardi et al., 2001b). At inhalation this “vagal break” is released and the heart accelerates because of a tonic sympathetic influence and the natural frequency of the sinoatrial node (Porges, 2011). RSA is the result of this on-off vagal activity. It has therefore been regarded as a measure of vagal tone (the parasympathetic effect on the heart mediated by vagus) (Hayano et al., 1991). It is documented that pronounced RSA is beneficial to circulation (Bernardi et al., 1998, 2001a; Friedman and Coats, 2000) and improves wellbeing (Friedman and Coats, 2000), whereas uneven or reduced RSA is a predictor of circulatory complications (La Rovere et al., 1998). For this reason yoga breathing and guided breathing (which both produce pronounced RSA) have beneficial effects on blood pressure and HR (Pramanik et al., 2009; Sharma et al., 2011).

Bernardi et al. have shown that reciting the rosary prayer enhances cardiovascular rhythms such as diastolic blood pressure and HRV (Bernardi et al., 2001c). They also find that the reading of one cycle of the rosary takes approximately 10 s (0.1 Hz) and thus causes readers to breathe at 10 s intervals. Bernardi and colleagues reveal that RSA is dependent on the respiration rate; it is more marked at about 0.1 Hz respiration (Bernardi et al., 1989) and baroreflex sensitivity is enhanced at the same breathing frequency (Bernardi et al., 2001a). Other studies, however, show that RSA can be just as marked at lower frequencies (Nesterov et al., 2005).

Müller et al. find that phase synchronization of respiration and HRV increase significantly in between subjects singing in unison and in canon (Muller and Lindenberger, 2011). This is the first article to describe inter-individual synchronization of cardiac and respiratory patterns caused by singing.

We believe that the reason for the observed HRV entrainment in Müller's study is to be found in the fact that unison singing coordinates respiration with consecutive RSA effects.

So far, the few studies performed on choir singing have used existing choir music. Here, we use a mantra, composed with a regular short theme to be repeated over and over and streamlined to accentuate HRV and RSA. We thus test song-phrases (expiration) that together with inspiration (inhalation) encompass 10 s. Hereby the mantra guides a respiratory cycle of 0.1 Hz. This is compared to humming where participants are asked to keep one tone and inspire independently whenever they want to. This condition thus contains unsynchronized singing. We compare these simple song structures to ordinary singing, where the choir sings a Swedish version of the international hymn *Fairest Lord Jesus (Härlig är jorden)*. To enable a more in-depth examination of the interrelation between HR and respiration, we complement the group study with five case studies using more advanced equipment for simultaneous recording of HR, respiration, finger temperature and skin conductance. The hypothesis for this study is that the three different song structures will cause three different respiration patterns which, due to RSA, will cause tree different HRV patterns.

## Materials and methods

This study is ethically approved by *Regionala Etikprövningsnämnden i Göteborg*. Informed consent has been obtained from all subjects.

### Group study

The group study comprises fifteen healthy 18 year olds of mixed gender. The subjects perform three singing tasks as a choir. The three different song structures we examine (hum, hymn, and mantra) are each of 5 min duration, separated by a 1 min pause during which instructions for the next singing task are recapitulated. Below, we summarize the study design.

**Figure 1:**
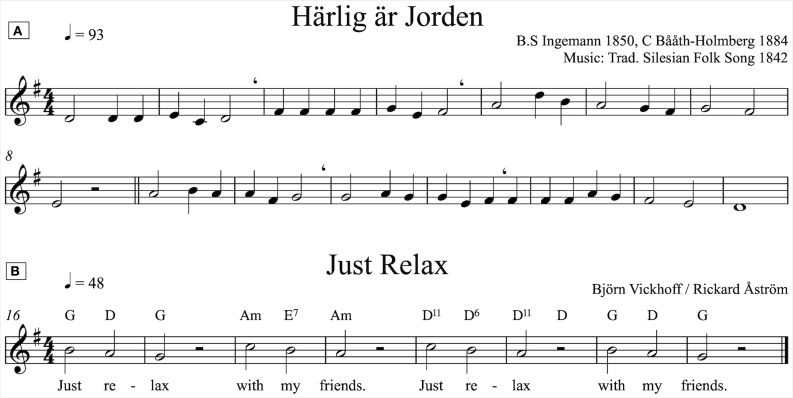
**(A)** The hymn *Fairest Lord Jesus* (Swedish *Härlig Är Jorden*). The tempo 93 bpm means that two bars take 5.156 s, which invites singers to a 0.194 Hz respiration rate. Four bars take 10.312 (0.097 respiration rate). **(B)** The mantra. Singers are asked to breathe solely between the phrases, which corresponds to a respiration rate of 0.1 Hz.

1. 0–5 min: subjects silently read an emotionally neutral text (baseline).
2. 5–10 min: subjects hum (keep a collective tone). The humming implies holding a tone (no text) and breathing whenever needing to.
3. 11–16 min: subjects sing *Fairest Lord Jesus* (Figure 1A). This song was chosen because it is well known and is a representative hymn in terms of tempo and structure. The singers, who have access to the lyrics but not to the score, are accompanied by a pianist.
4. 17–22 min: subjects sing a 10 s phrase mantra *Just Relax* (Figure 1B). The mantra, composed for the occasion, contains song phrases of 10 s duration, producing respiration cycles of 0.1 Hz (including inspiration). Subjects are strictly instructed to inspire only between phrases. The singers are accompanied by a pianist.
5. 23–28 min: subjects silently read an emotionally neutral text (baseline).

The above design makes it possible to compare the singing of a mantra, composed to produce RSA, to non-synchronized singing (humming) and ordinary singing (the hymn). The silent reading is the baseline condition.

The music has three levels of temporal structure:

1. Humming has no temporal structure.
2. The mantra has a two bar structure (Figure 1B).
3. The hymn has a hierarchical structure (Figure 1A). 16 bars are divided into two 8 eight bar sequences. These sequences are in turn divided into four bar sequences, which sometimes consist of two bar modules. This structure is indicated by the melody, pauses and breathing instructions.

The design thus allows for an examination of three different levels of respiratory coordination. Humming is not coordinating; the mantra is completely coordinating, since the structure as well as the instruction leaves no room for individual choice; the hymn is coordinating to some degree, since the structure of the song imposes a preferred respiration.

The HR activity of the group subjects (15 healthy 18 year old subjects of mixed gender) are recorded using ear clips for optical reading (emWave technique developed by HeartMath, www.heartmath.org). This equipment allows us to assess HR for all participants simultaneously. During HR recording, subjects sit in a semi-circle with their eyes open. Although all subjects have previous experience of choir singing, the group rehearses the singing tasks prior to HR activity recording. Due to loss of signal and/or alignment difficulties, four recordings are excluded from the analysis.

### Case studies

To obtain a more exact HR recording, paired with respiration and ANS activity, we also conduct five case studies measuring: HR, respiration depth and frequency, skin conductance level, and finger temperature. The data is collected using cStress (developed by PBM Stressmedicine Systems AB). cStress has precise timing capability which allows for HRV phase calculations between subjects.

The five subjects perform the three singing tasks together, just as in the group study. For technical reasons, cStress can only record data for one subject at a time. Thus, subjects take turn to be the recorded while singing or to be a choir participant (meaning the full study, identical to that of the group study, was repeated five times). The precise time to switch between conditions (see items 1–5 of the study design above) are pre-coded into cStress and thus the separate recordings are perfectly aligned between the five different subjects.

### Statistical methods

The emWave device detects the heart beats via an optical reader attached with a clip to the earlobe. The sampling rate is 250 Hz and the emWave stores the results as inter-beat intervals (IBI). For HRV assessment and related measures we followed the recommendations in Task Force of the European Society of Cardiology the North American Society of Pacing Electrophysiology (Malik, 1996).

The IBI are transformed to instantaneous estimates of HR, observed at the time stamp of each recorded heart beat and interpolated in between those time stamps. The individual instantaneous HR recordings are aligned between subjects using the time stamps. These irregularly sampled HR time series are then resampled to a common 5 Hz sampling frequency from which HRV measurements are extracted. HRV spectral densities are computed in rolling windows of size 96 s at 12 s steps (spectral calculation using detrending, smoothing, tapering and padding) (see e.g., Brillinger, 2001). We also compute the spectral measures in each singing condition (5 min segments).

*HRV measurements* that we study include RMSSD and the LF/HF ratio (e.g., Malik, 1996). The LF component was defined as the power in the frequency domain 0.04–0.15 Hz and the HF component as the power in the frequency domain 0.15–0.4 Hz. However, both the RMSSD and the LF/HF ratio were found to be insufficient to distinguish between the four conditions in the study (baseline, humming, hymn singing, and mantra singing). We therefore also compared the spectral densities in full via frequency scores (within a subject) and coherence (between subjects).

*Frequency scores* are computed to summarize the regularity of the HR fluctuations. The frequency score for a time window is defined as the percentage area under the spectral density in a ±0.01 Hz range around the most pronounced or dominant frequency. That is, for a pure sinusoid HR fluctuation (HR acceleration/retardation at precise regular intervals) the frequency score is 1 (or 100%). For an irregular HR fluctuation, accelerating and slowing down at random, the frequency score is near 0.

Cross-spectral densities are computed between each pair of subjects. From these spectra the coherence and phase are computed. The coherence captures the co-variation at each frequency (i.e., to what extent a HRV frequency is common to two subjects). Coherence *p*-values are computed at each frequency (Brillinger, 2001) and the most significant coherence extracted. This constitutes the *coherence score*. The *phase score* is extracted from the phase spectrum at the frequency corresponding to the most significant coherence.

The analysis is also repeated for fixed sized time windows, comprising the central 4 min segments of each condition (hum, hymn, and mantra). The 30 s beginning and end of each segment was omitted to avoid bias due to partial overlap of responses from another singing task. Frequency scores are statistically compared between conditions using Analysis of Variance (ANOVA) with multiple comparison corrections (there are six paired comparisons between baseline, hum, hymn, and mantra). ANOVA is also used to compare RMSSD between conditions. The significance analysis is performed on (1) scores computed from the central 4 min segments of each condition and (2) from average scores from rolling windows located in each condition. Conclusions do not differ between the two forms of analyses. Coherence and phase scores are compared using ANOVA with effective sample size given by the number of subjects. For the phase scores a third form of analysis is added: (3) ANOVA on the standard deviation of the phase scores from rolling windows in each condition. Analysis (3) is used to test *phase lock*, i.e., that the phase shift between subjects holds steady (does not vary) within a condition.

RSA is computed as the most significant coherence between respiratory depth and HR and compared across conditions using ANOVA with multiple comparison corrections.

All *p*-values reported below are adjusted for multiple comparisons.

## Results

### Group study results

Of the 15 subjects who participate in the study, four recordings are not included in the analysis due to technical reasons (discontinuous registration). The sample size is this *n* = 11 for the group study.

**Figure 2:**
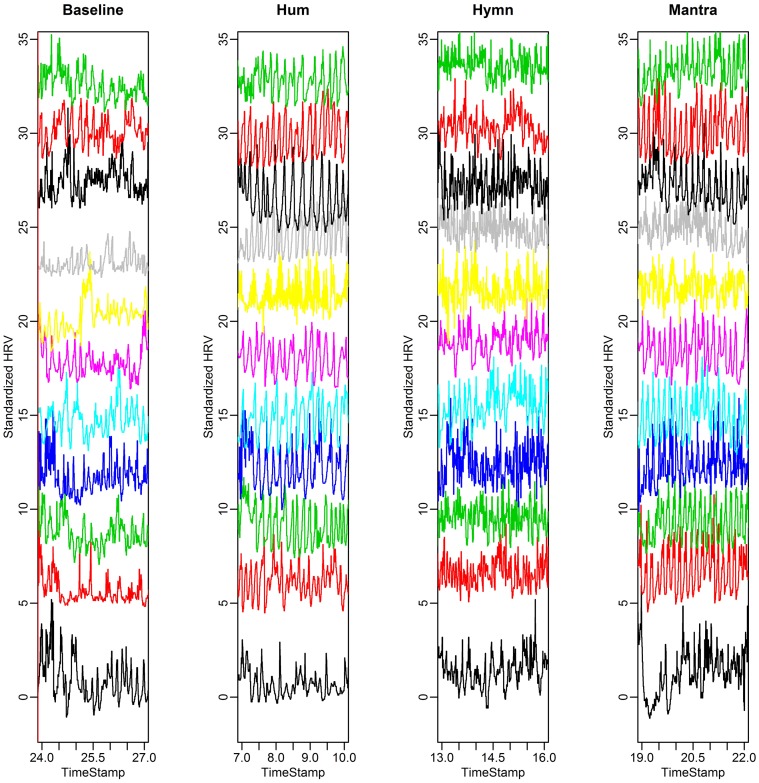
**HR curves for the eleven subjects in the group study**. Each HR curve is standardized and plotted at different mean levels to simplify visual comparisons between subjects. We depict 3 min excerpts from each condition (baseline, hum, hymn, and mantra).

The fluctuations in HR over time are shown in Figure 2. This is thus a visual presentation of HRV. HRV is first calculated with RMSSD (Root Mean Square of the Successive Differences). Analysis of Variance (ANOVA) indicates that the hymn and the mantra conditions induce significantly higher RMSSD than the humming and baseline conditions (*p*-values < 0.05 after multiple comparisons correction). We also compared the LF/HF ratio between conditions. The LF/HF ratio could only distinguish the Hymn singing from the other three conditions (*p*-values < 0.05). We conclude that the LF/HF ratio for vagal tone cannot be used because of the design of the experiment. The 0.1 Hz respiration reduces the HF HRV which will lead to an underestimation of the vagal activity.

**Figure 3:**
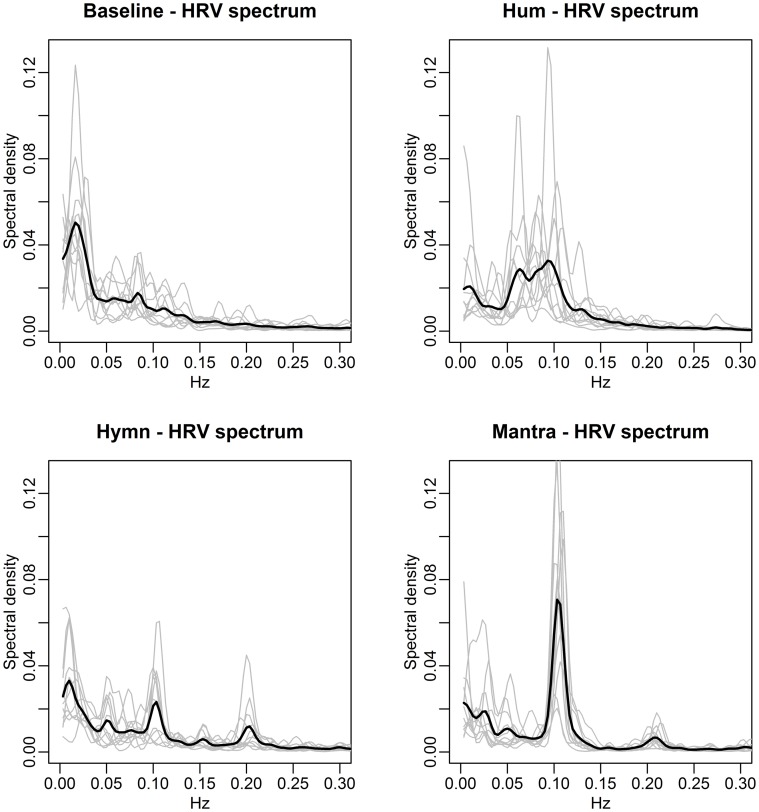
**The four conditions induce different HRV-frequencies**. Each panel depicts the individual subjects' spectral densities (gray curves) in the four different conditions. The black lines represent the mean spectral densities across subjects. Baseline spectra **(top left)** exhibit no dominant frequencies. Humming **(top right)** results in each individual spectra containing a clear dominant frequency, albeit at different frequencies for different subjects, reflecting subjective choices of breathing pauses. As a result, the mean spectral density does not contain a clear dominant frequency but rather a range from 0.05 to 0.1 Hz. Singing of the hymn **(bottom left)** results in individual spectra that are more similar in nature, with dominant peaks at frequencies 0.05, 0.1, and 0.2 Hz (corresponding to song phrases, Figure 1A). The individual spectra for mantra singing **(bottom right)** clearly show that the HR fluctuates at 0.1 Hz for all subjects.

HRV is further examined via frequency domain analysis. Spectral densities are computed from the central 4 min recordings of each condition and shown in Figure 3.

The spectral densities of individual subjects in the four conditions clearly indicate distinctly different frequency patterns:

1. Baseline: as expected, each individual spectrum contains no dominant HRV frequency (the higher values for very low frequencies simply indicate the presence of a slow varying trend in overall HR).
2. Humming: each individual exhibits a dominant HRV frequency, but these differ from individual to individual, although lie approximately within the span of 0.05–0.1 Hz. The mean spectral density (black line) is thus quite flat since the subjects' HR curves do not share a dominant frequency of variation.
3. Hymn: each individual spectrum is dominated by HRV frequencies at 0.1 and 0.2 Hz.
4. Mantra: the structured song phrases of the mantra results in an unambiguously focused and highly regular HRV frequency at a 0.1 Hz for all individuals.

The spectral densities are summarized as frequency scores, defined as the percent of the density within ±0.01 Hz of the dominant frequency. These scores are then compared across conditions via ANOVA with multiple comparisons correction. This reveals no significant difference between the baseline and hymn conditions and no significant difference between the humming and mantra conditions, but a significantly higher frequency score (more regular HR variation) in mantra and hum compared with baseline and hymn (*p*-values < 0.01 after multiple comparisons correction). We can thus conclude that humming as well as mantra singing produce very regular variations in HR, but this is not the case for ordinary (hymn) singing (or, as expected, during baseline).

Coherence is a frequency domain statistic that summarizes the co-variation (correlation) of two subjects at each frequency. A high average coherence at a certain frequency indicates many subject pairs have HR curves that vary at this common frequency.

**Figure 4:**
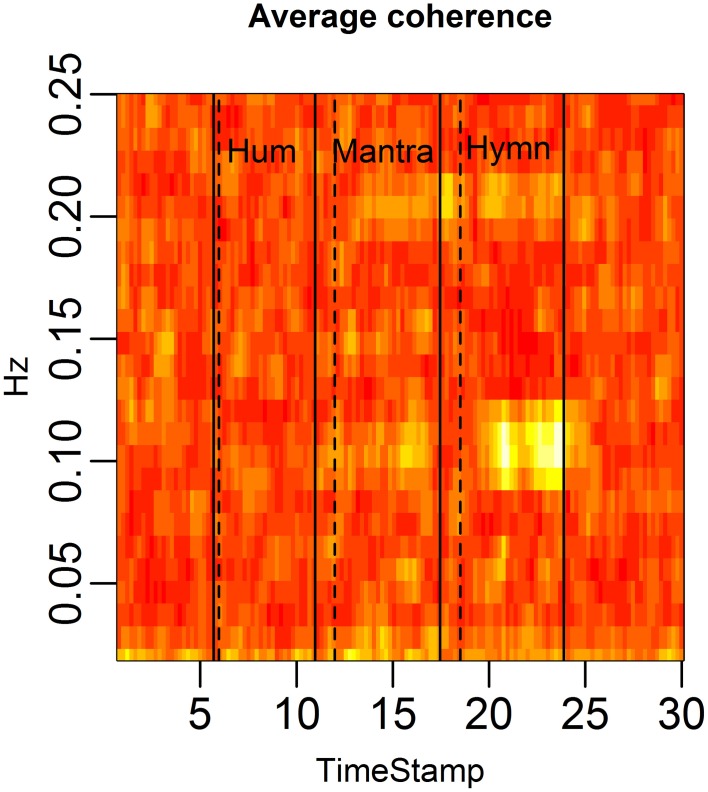
**HRV between-subject coherence**. Each column of the figure represents the average coherence across pairs of subjects for a certain time window. Each row represents a frequency in Hz. The coherence is computed in rolling windows of length 96 s, step size 12 s. The coherence summarizes the co-variation (correlation) of two subjects per frequency. In the figure, brighter colors represent higher coherence. Coherence is clearly higher during the mantra than during any other condition (0.1 Hz). Coherence is also higher during the hymn than during humming and baseline.

Figure 4 demonstrates the distinct patterns in coherence for different singing conditions:

1. Baseline: there is no significant coherence in HRV between individuals [concluded from the *p*-values from each subject pair's coherence at each frequency (Brillinger, 2001)].
2. Humming: we know from the results in Figure 3 that humming produces regular HR fluctuation, i.e., HRV spectra with dominant frequencies, but since these frequencies are different for different individuals, there is no significant coherence. This is also apparent from the panel marked “Hum” in Figure 4.
3. Hymn: although Figure 3 indicates that each individual frequency spectrum is not as strongly dominated by a single frequency as during the humming, coherence analysis reveals that subject pairs significantly share HRV frequencies. As can be seen from Figure 4, the most shared frequencies are around 0.1 and 0.2 Hz. This corresponds to the peaks of the spectral densities in Figure 3 (bottom left panel).
4. Mantra: the coherence is highly significant in the mantra for the 0.1 Hz field, which is to be expected from the results in Figure 3.

We summarize our findings from the group study here. While humming does not produce a significant increase in HRV as measured by RMSSD, we can conclude that humming does lead to a significantly more regular HRV (0.05–0.1 Hz) as measured by the frequency score (*p*-value < 0.01). That is, HR acceleration and deceleration is quite regular during humming, although the rate of the HR fluctuation is highly individual. This is statistically verified by the non-significant coherence between the humming segment HR curves of subject pairs.

HRV as measured by RMSSD is significantly increased during hymn singing compared to both baseline and humming (*p*-value < 0.05), and also exhibited significantly lower LF/HF ratio compared with the other conditions (*p*-value < 0.05). However, frequency analysis indicates that HR fluctuations are not as regular as during humming (*p*-value < 0.01). Interestingly, despite the less obvious regularity of the HRV during hymn singing, coherence analysis shows that HR fluctuations occur at common, shared frequencies for subjects (predominantly 0.1 Hz). The coherence is significantly increased compared to both baseline and humming (*p*-value < 0.05).

Finally, the mantra produces a significantly higher RMSSD compared to all other conditions (*p*-value < 0.01) as well as significantly more regular HRV (frequency score) compared with baseline and humming (but not hymn singing) (*p*-value < 0.01). The coherence analysis clearly shows that mantra singing induces a strong, shared HRV frequency component at 0.1 Hz, significantly higher compared to all other conditions (*p*-value < 0.01). [All *p*-values in this section are corrected for multiple comparisons (the six pairwise tests between four conditions)].

Together, the results indicate a strong connection between song structure and HR patterns.

### Case study results

The results of the group study demonstrate that there is a connection between the length of the song phrases and HRV frequencies. A possible explanation for this is RSA (i.e., that HRV is coupled to respiration).

To investigate this further, we examine five subjects (who did not participate in the first study) in a separate case study. Using more advanced equipment (cStress), we simultaneous record HR and respiration depth for all subjects during the three singing tasks previously described. While the recordings are conducted on one subject at a time, the subjects are still singing with others and follow the same succession of conditions as in the group study. cStress allows for more precise time readings compared with the portable emWaves used in the group study. This enables phase estimation of the HRV graphs. Finger temperature and skin conductance are also measured, but these do not produce any significant results.

**Figure 5:**
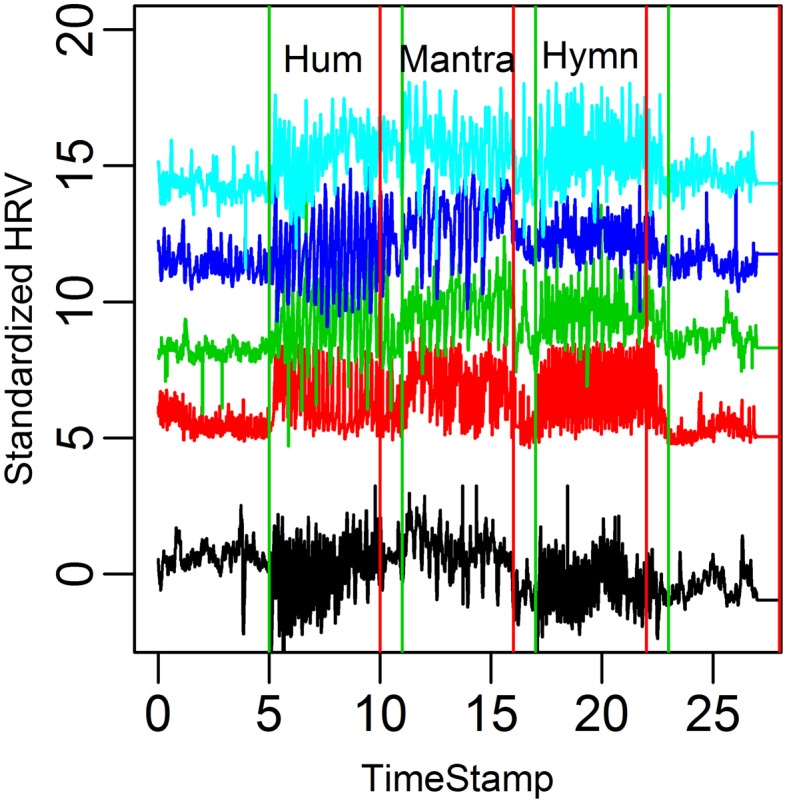
**HR graphs for the five subjects in the case study over the entire time domain**.

The HR variation for the five subjects is depicted in Figure 5.

A visual inspection suggests that HRV increases in all conditions compared to baseline. Because of the small sample size, however, comparison in terms of RMSSD and LF/HF across conditions results in *p*-values ~0.1 (after multiple comparisons correction).

**Figure 6:**
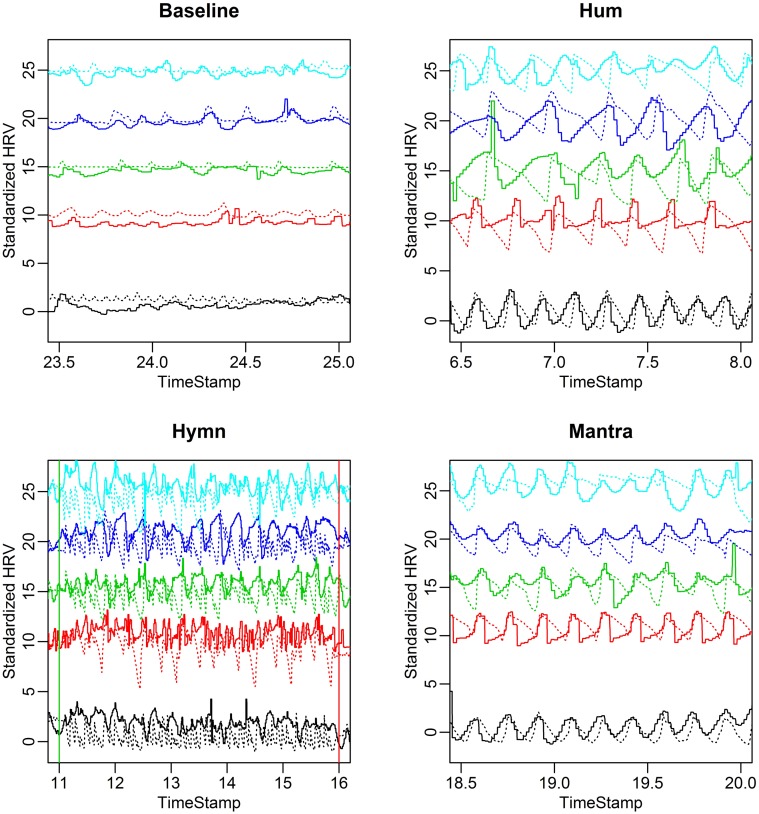
**HR (solid curves) and respiration depth (dotted curves) for the five different subjects in the case study**. Both HR and respiration depth are standardized to have standard deviation equal to 1. This makes HR and respiration curves comparable in the same plot and relative amplitude variation in different conditions become apparent. Each panel depicts a representative 90 s interval for each of the four conditions. Notice the subject-to-subject variability in terms of both respiration and HRV frequency during humming **(top right)** compared with a highly synchronized breathing and HRV pattern during the mantra **(bottom right)**. The breathing pattern of the hymn **(bottom left)** is clearly more complex, comprising phrases of different length (0.05, 0.1, and 0.2 Hz, cf. Figure 1B). During baseline **(top left)** HR and respiration depth exhibit overall lower amplitude of variation.

To illustrate the coupling between HRV and respiration, we depict both HR and respiration depth curves for representative 90 s intervals from each of the four conditions (Figure 6).

A clear pattern emerges:

1. Baseline exhibits low HR variation and respiration depth and possibly a weak alignment between the two.
2. HR and respiration curves extracted from the humming segment demonstrates that the instruction to hold a tone results in subjects breathing in considerably longer cycles and much more deeply. The coupling between HR and respiration is apparent.
3. Hymn singing produces high amplitude HRV and respiration but the variation is less regular compared to humming. The respiration and HR do not appear to be strongly phase locked.
4. The mantra exhibits a clear respiration and HR curve alignment in terms of amplitude, frequency and phase.

**Figure 7:**
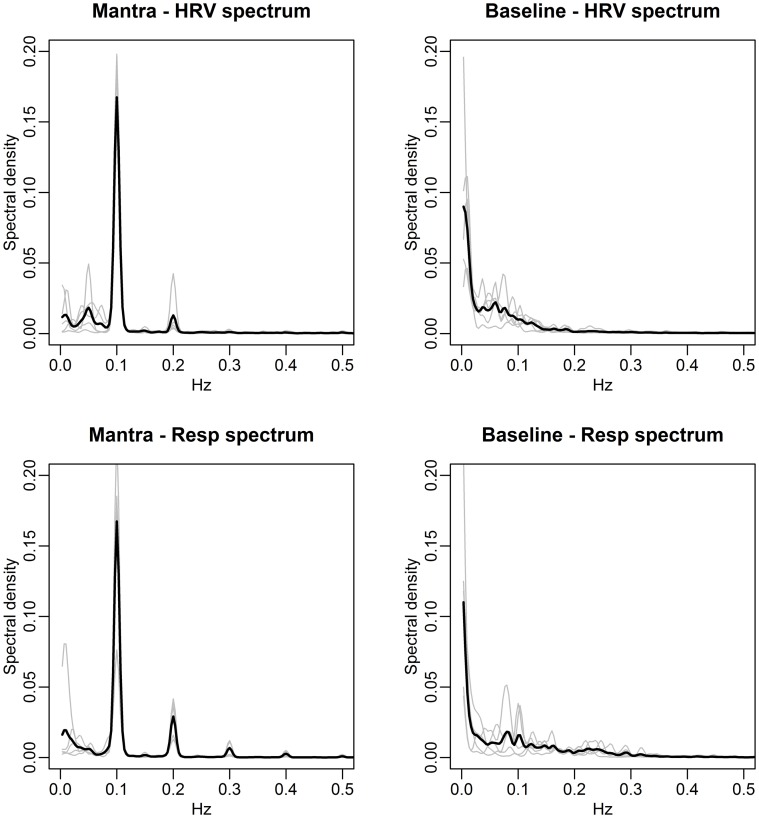
**Spectral densities of HRV (top) and respiration depth (bottom) for the Mantra (left) and Baseline (right) conditions (case study)**. The individual spectra are depicted as gray curves and the mean spectral density across subjects is shown in black. Notice how both the HRV and the respiration depth during mantra singing are dominated by 0.1 Hz fluctuations for all individuals (the 0.2 Hz is a harmonic due to the non-sinusoidal nature of the fluctuation, cf. Figure 6).

**Figure 8:**
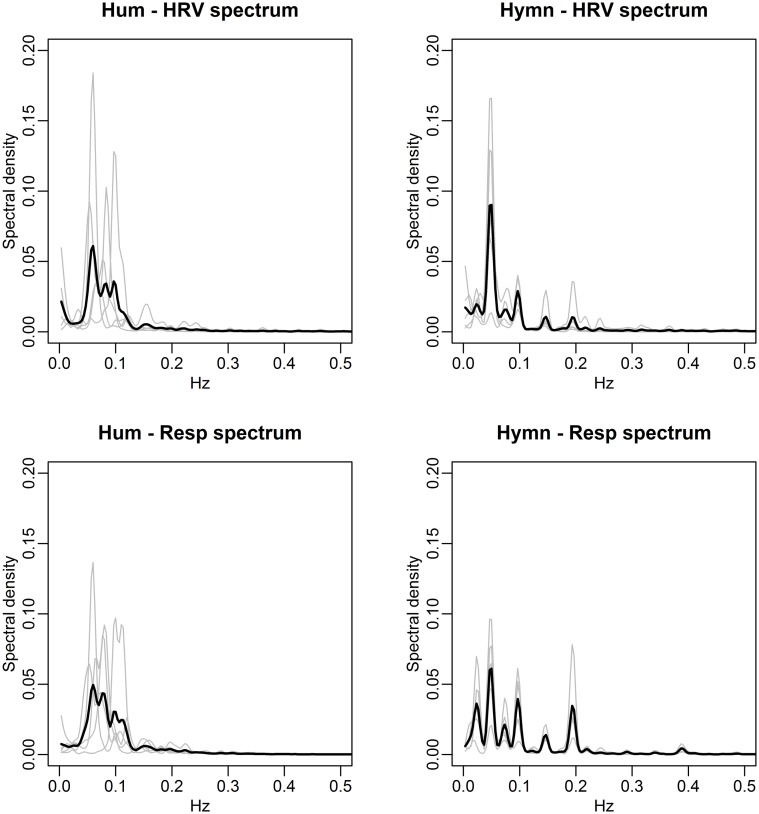
**Spectral densities of HRV (top) and respiration depth (bottom) for the Hum (left) and Hymn (right) conditions (case study)**. The individual spectra are depicted as gray curves and the mean spectral density across subjects is shown in black. The HRV and respiration depth spectra for humming clearly illustrate the subjective choices of breathing pattern and its impact on HR (left panels). There is more subject-to-subject commonality during the hymn (right panels), with respiration cycles at 0.05, 0.1, and 0.2 Hz (bottom right). The HRV spectra (top right) are also dominated by the 0.05, 0.1, and 0.2 Hz frequencies (cf. Figure 9).

We conduct frequency domain analysis allowing HRV frequencies to be compared to respiration frequencies (Figures 7, 8).

The agreement between the HRV frequency spectra and the respiration frequency spectra is striking. This is thus a visual illustration of RSA. Significance analysis of the frequency scores indicate that HRV fluctuations are significantly more regular during any form of singing compared with baseline (*p-value* < 0.01). Due to the small sample size we cannot distinguish between frequency scores in the different conditions. As in the group study, Figure 7 (left panels), clearly shows that HR fluctuates at 0.1 Hz for all individuals during mantra singing. The bottom panel confirms that this is likely a result of the guided respiration at 0.1 Hz common to all subjects. Figure 8 (left panels) shows that humming results in individual HRV spectral densities that contain a dominant frequency in the 0.05–0.1 Hz range, different for each subject. The likely cause is again apparent from the respiration spectra which summarize the individual breathing patterns. The right panel of Figure 8 shows that HRV induced by hymn singing is dominated by a 0.05 Hz frequency, with smaller peak frequencies at 0.1 and 0.2 Hz. The respiration spectra exhibit the likely underlying cause: a respiration pattern at 0.05 Hz, as well as at 0.1 and 0.2 Hz. The peak at 0.05 Hz in hymn respiration (Figure 8, bottom right) may seem puzzling, since it indicates a respiration cycle of 20 s. However, inspection of the music score reveals that the overall structure of the hymn is two sections of eight bars. Each section ends with a long phrase and a pause, meaning that singers have a long exhalation followed by a forcing opportunity to inhale. This 0.05 Hz peak is also discernible in the group study (Figure 3), although it is not as pronounced.

**Figure 9:**
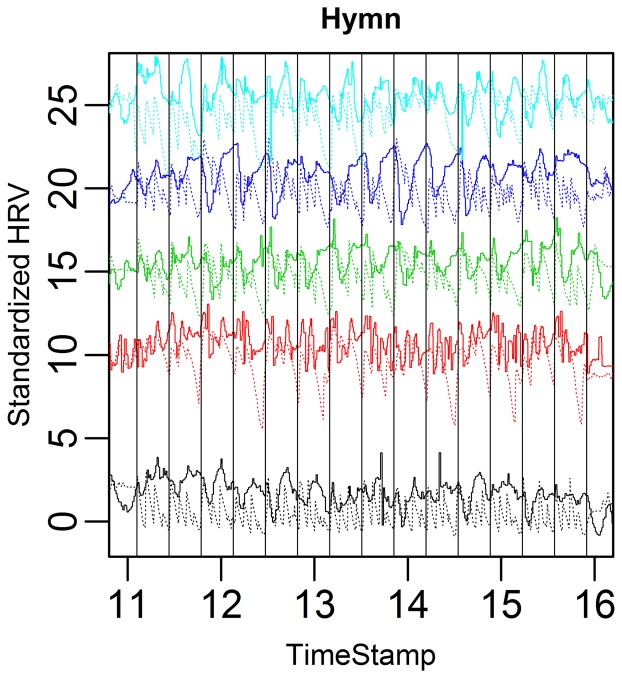
**Complete HRV (solid) and respiration depth (dotted) curves for the five case subjects during the hymn**. Verticals lines indicate the start of music score bar one and the start of bar nine. The song phrase respiration cycles are easily discernible (two, four, and eight bar cycles).

Figure 9 clearly shows that the subjects tend to exhale deeply at the end of each eight bar section (because of the antecedent long song phrases). This should thus not be interpreted as 20 s respiration cycles, but rather that this deep breath created an overarching respiration structure which reflects the overarching structure of the hymn. We can also see that the respiration/HRV connection is not so strong in the hymn. The impression is that the heart cannot completely follow the respiration when it has a more complex, hierarchical structure.

**Figure 10:**
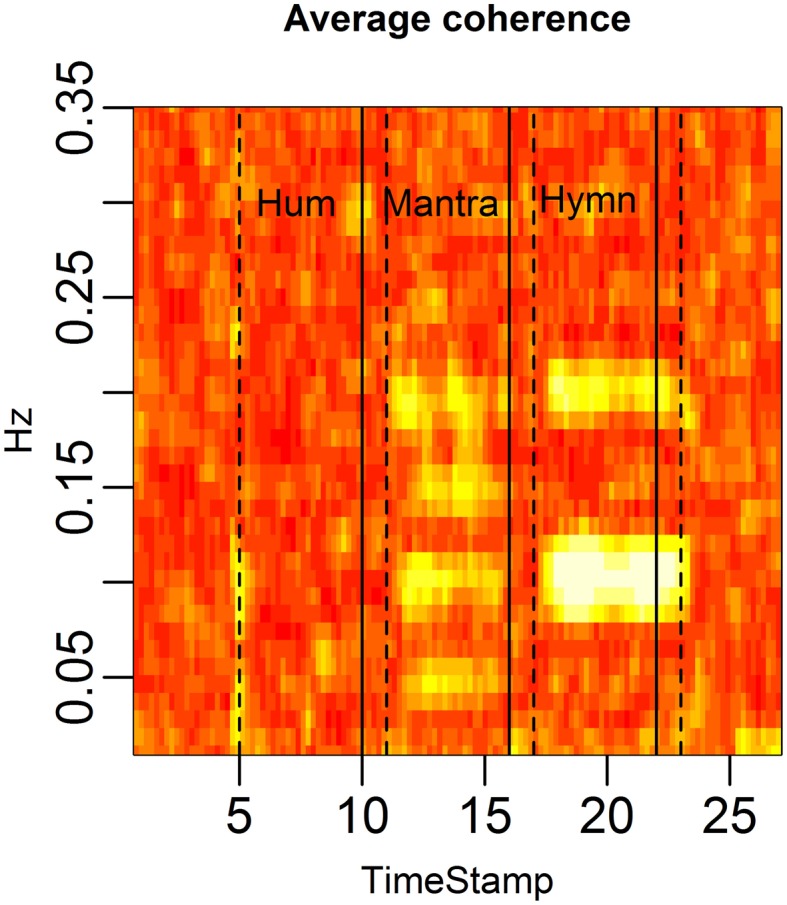
**HRV coherence for the case study**. Each column of the figure represents the average coherence across pairs of subjects for a certain time window. Each row represents a frequency in Hz. The coherence was computed in rolling windows of length 96 s, step size 12 s (cf. Figure 4). Coherence is clearly high during the mantra (at 0.1 Hz and at the harmonic frequency 0.2 Hz). There is also high coherence during the hymn (at 0.05, 0.1, and 0.2 Hz, and the harmonic 0.15 Hz).

The findings in Figure 9, connecting biological patterns to music patterns, are further illuminated in a coherence analysis depicted in Figure 10.

The structure of the musical score is reflected in the coherence. The dominant HRV frequencies that are shared across subjects show up as high coherence. In the mantra the 48 bpm tempo leads to 0.1 Hz breathing which causes 0.1 Hz HRV among all five individuals. This results in strong coherence in the 0.1 Hz frequency band. In the hymn we also see a significant (but weaker) coherence at 0.05, 0.1, and 0.2 Hz. The hymn has phrases of two and four bars which lead to 0.2, 0.1 respiration cycles. The deep breath every 20th second evidently causes a 0.05 HRV frequency that is shared across singers. Humming does not have a music structure and evidently does not produce a shared HRV frequency pattern.

**Figure 11:**
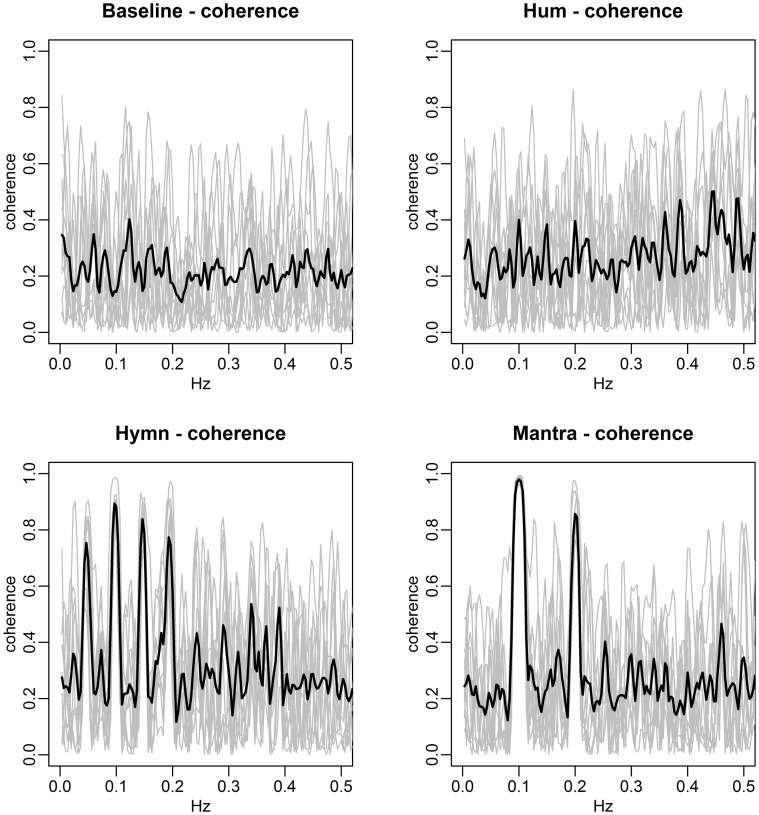
**HRV coherence per condition for the case study**. Each panel depicts the coherence as a function of frequency in a condition (gray curves represent the individual coherence, black lines the average across subject pairs). The hymn has phrases of two, four, and eight bars which lead to 0.2, 0.1, and 0.05 Hz respiration cycles (clearly seen in Figure 9), which in turn causes HRV of dominant frequencies. The dominant frequencies that are shared across subjects show up as coherence, particularly at 0.1 Hz as seen in the bottom left panel. (Due to the non-sinusoidal appearance of the HR fluctuations (Figure 9), harmonics at 0.15 Hz are also present.) In the mantra, the coherence is high at 0.1 Hz (and at the 0.2 Hz harmonic due to non-sinusoidal HR fluctuations). There is no strong coherence at any frequency during the hum and baseline conditions.

Figure 11 provides a more detailed view. The coherence of each subject pair is depicted for each of the four conditions. Clearly, there is no significant coherence in either baseline or during humming, while coherence is significantly higher during both hymn and mantra singing (*p*-value < 0.01). The coherence is also significantly higher in mantra singing compared with hymn singing (*p*-value < 0.05). All *p*-values are corrected for multiple comparisons.

**Figure 12:**
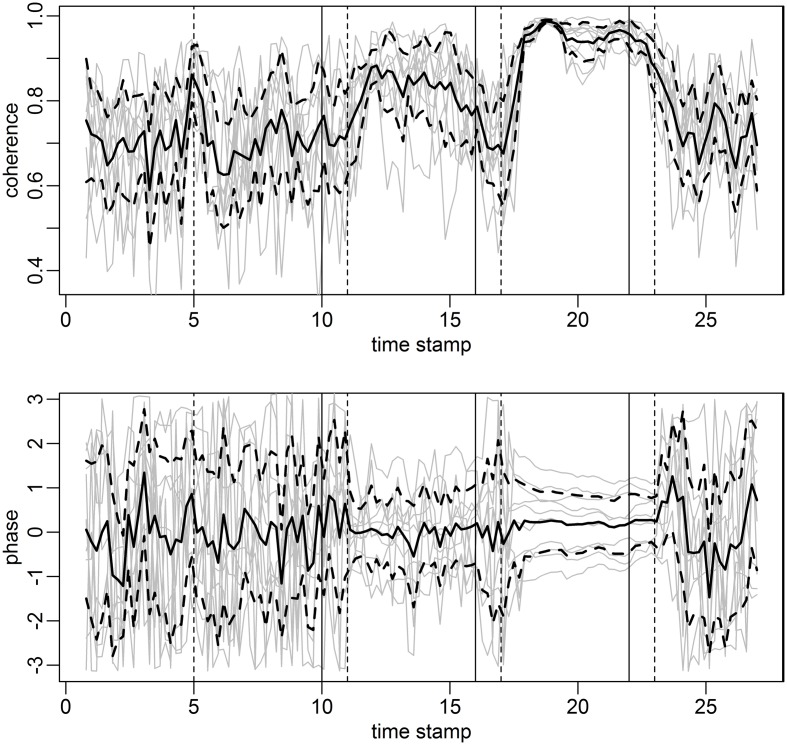
**Coherence and phase score curves for individuals in gray and mean across subjects in solid black**. The coherence score at each time point and for each pair of subjects (a point on the gray curve) is computed as the most significant coherence in each rolling window of length 96 s, stepped by 12 s. The phase lag at the frequency of the most significant coherence constitutes the phase score. Confidence bands (black dashed curves) are constructed from the individual score curves (gray) with effective sample size *n* = 5 (number of cases in the study). The coherence score **(top panel)** is clearly high during the mantra and hymn. There is a steady and decreasing phase lock during the mantra and to some extent during the hymn **(bottom panel)**.

We assess HRV phase next (Figure 12).

The top panel of Figure 12 simply illustrates the coherence score evolution for the duration of the study. The phase score is computed as the phase between subject pairs at the frequency of the most significant coherence. Phase 0 corresponds to a completely synchronized HRV, whereas phase ± π corresponds to a phase lag of a half-cycle. Figure 12 clearly shows that phase alignment is not present during humming or baseline. Hymn and mantra singing appears to be linked to somewhat smaller phase shifts (not significant due to the small sample size, *p*-value ~0.1). However, the striking result is rather the *phase lock* present during mantra singing. That is, the variation in phase shift between each subject pair decreases significantly during the mantra condition as compared to baseline and humming (*p*-value < 0.01). The phase variation during hymn singing is also significantly lower compared to baseline and humming (*p*-value < 0.01) but significantly higher compared with the mantra (*p*-value < 0.05). Visually, the phase lock can be deduced from the near parallel horizontal phase score curves during the mantra. There is possibly a slight tendency that the phase scores are approaching zero (no phase shift), thus suggesting that if we had prolonged the mantra singing all the hearts would have been totally synchronized in HRV phase and frequency. Interestingly, this tendency continues 1 min after the singers have stopped singing, to be brutally interrupted when the subjects start reading in the baseline condition. This shows that music structure is paramount to HRV phase and that the hearts of the five participants tend to accelerate and decelerate simultaneously during hymn and especially during mantra singing.

**Figure 13:**
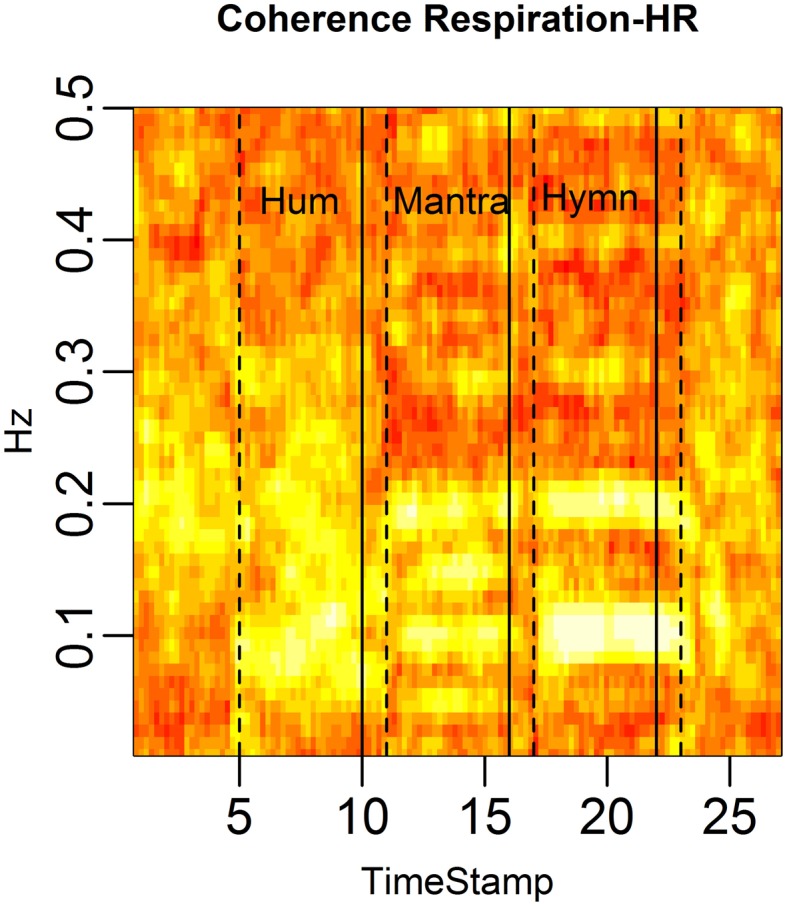
**RSA is defined as the coherence between respiration depth and HR**. We depict the average RSA across subjects in rolling windows of length 96 s, stepped by 12 s. Each column represents the coherence at different frequencies for a given time point and each row the coherence for a particular frequency across time. RSA is markedly high during the mantra (at 0.1 and the 0.2 Hz harmonic) as well as during the hymn (at 0.05, 0.1, and 0.2 Hz). RSA is also high during the hum segment, albeit not a common dominant frequency as expected since respiration frequency is highly individual during humming.

We have already seen how respiration and HRV are interdependent (RSA) (Figures 6, 9). Since the song phrases affect breathing and since breathing affects HR, the structure of the music affects HRV. One way to mathematically assess RSA is the estimation of HRV/respiration coherence. This is illustrated in Figure 13.

**Figure 14:**
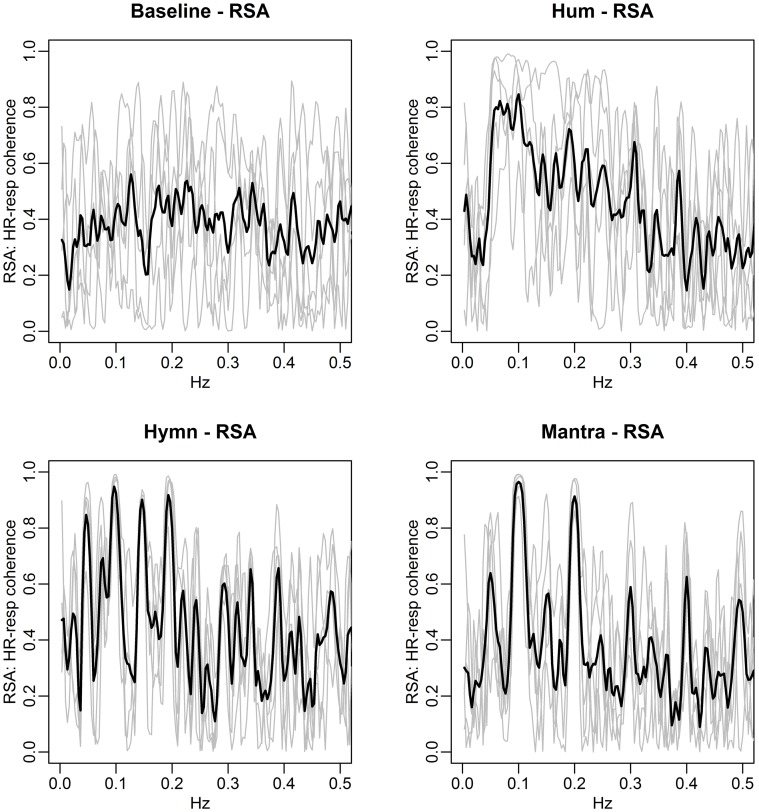
**HR-respiration coherence for individuals (gray curves) for each condition**. Average HR-respiration coherence is shown in black. RSA is defined as significant and high HR-respiration coherence. Notice the narrow frequency band for which RSA is observed during hymn and mantra singing. By contrast, humming is associated with RSA in a range 0.05–0.1 Hz and there is no clear RSA pattern during baseline.

The brighter fields in the figure show that RSA is strongest in the 0.1 Hz frequency during the mantra condition. The hymn condition shows weaker RSA but it can clearly be detected, mainly in the 0.05, 0.1, and 0.2 Hz fields, and the hum shows great variation but possibly that RSA is strongest just below 0.1 Hz which coincides with the mean respiration frequency for humming. When comparing RSA, as defined by the most significant coherence between HR and respiration, RSA is significantly higher during all singing conditions compared with baseline (*p-value* < 0.05). Due to the limited sample size, results are not significant when comparing singing conditions. Visual inspection of HR-respiration coherence, however, clearly indicates markedly different RSA patterns for the different song structures. This is especially evident from Figure 14.

The figure identifies the frequencies that are linked to RSA during each of the conditions. During baseline, RSA is lower than during any form of singing. Humming is associated with RSA in the range 0.05–0.1 Hz. By comparison, RSA is narrowly focused to particular frequencies for all subjects during hymn and mantra singing, predominantly at 0.1 Hz.

Taken together, these results show that the structure of the song determines respiration which in turn causes simultaneous acceleration and deceleration of the hearts of singers.

## Discussion

### Main findings

The group and case studies both suggest that singing increases HRV. For simple song structures, like humming and mantra chanting, the impact on HRV is especially marked in terms of high and regular amplitude variation (a dominant HRV frequency). Ordinary song structures, here represented by hymn singing, are observed to increase HRV as measured by RMSSD.

When the music (singing) structure is regular, HRV profiles tend to conform between singers in terms of frequency and phase. This is in agreement with Mûller's study reporting HRV frequency and phase compliance between singers.

Since music structure guides respiration for singers, the fact that HRV reflects music structure can be explained by RSA. Coherence analysis of HR and respiration indeed confirms that RSA mirrors music structure. This indicates that music structure determines respiration rates and that it is at these rates we can see respiration/HRV entrainment.

This means that there is a clear tendency toward an entrainment effect between singers in terms of HR acceleration and deceleration as soon as they sing a simple structure in unison. The underlying cause (RSA) is not totally understood but two dominating possibilities in the literature are mentioned in the introduction: interaction between pulmonary stretch receptors and the heart, and/or a cardiopulmonary oscillator consisting of interneurons connecting brainstem nuclei (the nucleus of the solitary tract and the nucleus ambiguous).

### Possible implications

As stated earlier, RSA is associated with vagal influence and self-reported well-being. Singing can be viewed as initiating the work of a vagal pump, sending relaxing waves through the choir.

Singing, and especially upbeat energetic singing, is at the same time arousing, since every activity and particularly physical activities are arousing. The assessment of finger temperature and skin conductance in this study did not, however, show significant sympathetic activity. This result could be interpreted as a vagal dampening of sympathetic activity. In other words, there was probably sympathetic activity but it was leveled out. The combination of sympathetic and vagal influence reflects the fact that these systems can act independently (Paton et al., 2005). It has been suggested that the mental state of *flow* (Csikszentmihalyi, 1997) may be such a combination (De Manzano et al., 2010). One of the characteristics of flow is focused interest during maintained control (Snyder and Lopez, 2009). This suggests sharp attention and action potential in a calm state.

Stephen W. Porges, argues in his *Polyvagal Theory* that social engagement demands a calm, unthreatened state combined with arousing motivation to participate (Porges, 1995). These functions, Porges emphasizes, are all associated with the myelinated vagus emanating from the nucleus ambiguus (Porges, 2011). As he also points out, this nucleus not only communicates with the heart but also with laryngeal vocal cord muscles via the recurrent branch of nervus vagus to the effect that ANS status is audible in human emotional prosody (Porges, 2003). Thus, music communicates the ANS state between singers in two ways: the vagal pump and an audible cue.

From the perspective of *joint action* (Sebanz et al., 2006) and entrainment, we claim that external and visible joint action corresponds to an internal and biological joint action. Entrainment between two dancers, for example, depends on analogue representations of rhythm (stimulated by the music) in their nervous systems. It is tempting to reverse this logic and consider how synchronized internal events affect external action. Choir singing coordinates the neurophysiological activity for timing, motor production of words and melody, respiration and HRV. It has been proposed that joint action leads to joint perspectives (Vickhoff, 2008) and joint intentions (Pacherie, 2011). In this context it is interesting to note that synchrony rituals benefit cooperation (Wiltermuth and Heath, 2009; David-Barrett and Dunbar, 2012). In other words, singers may change their egocentric perspective of the world to a *we-perspective* which causes them to perceive the world from the same point of view (of for example religion, politics or football team) and thus defining who *we* are.

This touches on the fundamental question of why music is a universal phenomenon. Unlike most other universal human behaviors there is no self-evident Darwinian explanation. The American cognitive musicologist David Huron, though, has proposed that music stimulates the production of the neuropeptide oxytocin and thus strengthens bonding (Huron, 2006). Since group bonding has a survival value, this might explain why we have music, he argues. The question of music and oxytocin is complicated. It is, due to the blood/brain barrier difficult to get direct indications of oxytocin in the brain. Venous oxytocin can be obtained, however. We have tested this in our laboratory for various types of music without indications of unambiguous oxytocin level changes. On the other hand, oxytocin level increase has been observed after singing lessons (Grape et al., 2003). Be it as it may, if collective singing creates joint perspectives, it would indeed be bonding in the deepest sense.

The vagal effect of breathing is, as pointed out, an ANS reaction. It is hardwired and thus universal. It could therefore be expected that various cultures use this technique wherever people gather to achieve relaxed communicative states. Interestingly, coordinated respiratory activity, irrespective if it is caused by yoga breathing, mantra chanting, praying or singing is ritually performed in most religions. This is a common factor, more so than the semantic content of beliefs.

### Limitations

The emWave tool used in the group study does not have the necessary time accuracy to determine phase in HRV. For this reason we could only show frequency compliancy but not determine phase compliancy. We therefore added a complimentary case study, which clarified the mechanisms and allowed us to follow respiration in combination with HRV.

Would the results in this study have been different if we had reversed the order of stimulus presentation? Since we had 1 min baseline (non-singing) between singing conditions, we could clearly see that there was no lingering HRV effect in the baselines from preceding conditions. The singing/HRV dependency presented in this study is very robust. We therefore draw the conclusion that the order of presentation would most likely not affect the results.

### Future research

This study consists of two parts: a group study and a case study. We did not make any comparisons between the group and the single cases. The case study involved taking respiration rate and HR measurements from single cases. However, the other case subjects were also participating in the singing during the measurements. In the future it would be very interesting to do test the hypothesis that there is a difference in neurophysiological variables if you sing alone or in a group.

Our study suggests that people who sing together tend to synchronize biologically in various respects. Eighty percent of the neural traffic between the heart and the brain goes from the heart to the brain. The natural question is how this affects the behavior of individuals and their perception of the world (during singing and after). Does choral singing produce a common perspective? How could such a perspective be manifested and measured? Another, quite different, question is if guided breathing could be associated to runners “second wind” (i.e., the observation that after a while of running, the running becomes more comfortable). Could it be that runners tend to synchronize respiration to the steps, or to imagery melodies which creates a slower paced breathing and thus affect HRV? This may make the circulatory system more efficient and thus produce distance or speed at lower energy costs.

So far, neuromusicology has mainly been concerned with cortical and limbic activity, but this study rather stresses the importance of the ANS system for music perception, production and communication. How music affects this system has broad implications for stress reduction therapy and motivation.

## Conclusion

We explain how the length of the song phrases guides respiration, resulting in compliances of frequencies and phases of respiration cycles and HRV cycles between singers. Singing produces slow, regular and deep respiration which in turn triggers RSA. This causes a pulsating vagal activity which, together with sympathetic activity, is interpreted in context of Porge's polyvagal theory of communication. The findings potentially explain the role of collective singing in the creation of joint perspectives.

### Conflict of interest statement

The authors declare that the research was conducted in the absence of any commercial or financial relationships that could be construed as a potential conflict of interest.
