# Erratum: Music structure determines heart rate variability of singers

**DOI:** 10.3389/fpsyg.2013.00599

**Published:** 2013-09-05

**Authors:** Björn Vickhoff, Helge Malmgren, Rickard Åström, Gunnar F. Nyberg, Seth-Reino Ekström, Mathias Engwall, Johan Snygg, Michael Nilsson, Rebecka Jörnsten

**Affiliations:** ^1^Center for Brain Repair and Rehabilitation, Institute of Neuroscience and Physiology, Sahlgrenska Academy, University of GothenburgGothenburg, Sweden; ^2^Department of Philosophy, Linguistics and Theory of Science, University of GothenburgGothenburg, Sweden; ^3^Professional Musician and ComposerTorslanda, Sweden; ^4^Department of Clinical Physiology, Sahlgrenska University HospitalGothenburg, Sweden; ^5^CantorKalvshult, Sweden; ^6^Department of Cultural Sciences, University of GothenburgGothenburg, Sweden; ^7^Department of Anaesthesia and Intensive Care, Sahlgrenska University HospitalGothenburg, Sweden; ^8^Hunter Medical Research Institute, University of NewcastleNewcastle, NSW, Australia; ^9^Department of Mathematical Sciences, University of Gothenburg and Chalmers University of TechnologyGothenburg, Sweden

**Keywords:** choral singing, heart rate variability, respiratory sinus arrhythmia, frequency analysis, autonomic nervous syste

## Erratum

Figures 4, 5, 10, 13 in the article “Music structure determines heart rate variability of singers” by Vickhoff et al. published in Frontiers in Psychology, 09 July 2013 (doi: 10.3389/fpsyg.2013.00334) contain a labeling error: The singing task tags “Hymn” and “Mantra” appear in the wrong order.

**Figure 4:**
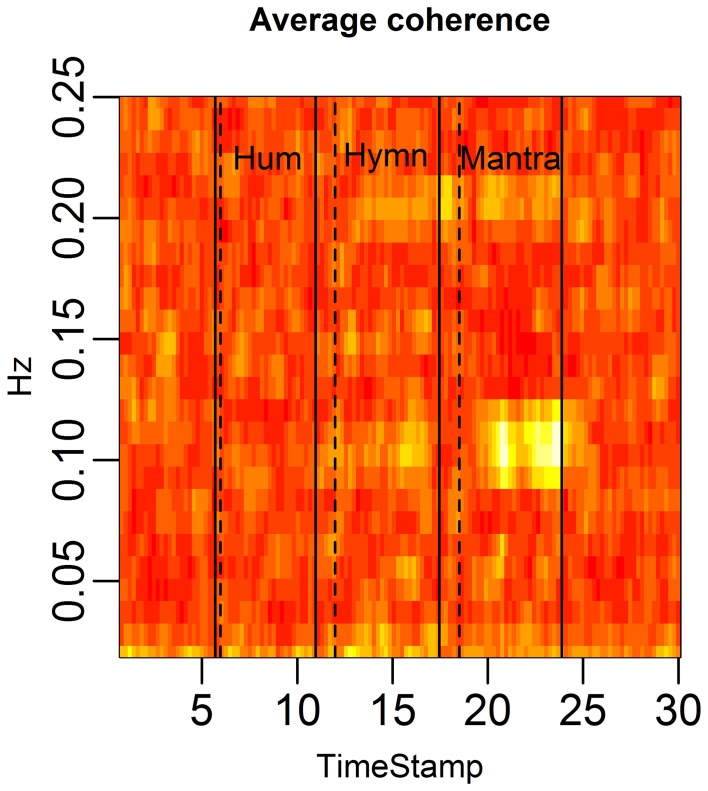
**HRV between-subject coherence.** Each column of the figure represents the average coherence across pairs of subjects for a certain time window. Each row represents a frequency in Hz. The coherence is computed in rolling windows of length 96 s, step size 12 s. The coherence summarizes the co-variation (correlation) of two subjects per frequency. In the figure, brighter colors represent higher coherence. Coherence is clearly higher during the mantra than during any other condition (0.1 Hz). Coherence is also higher during the hymn than during humming and baseline.

**Figure 5:**
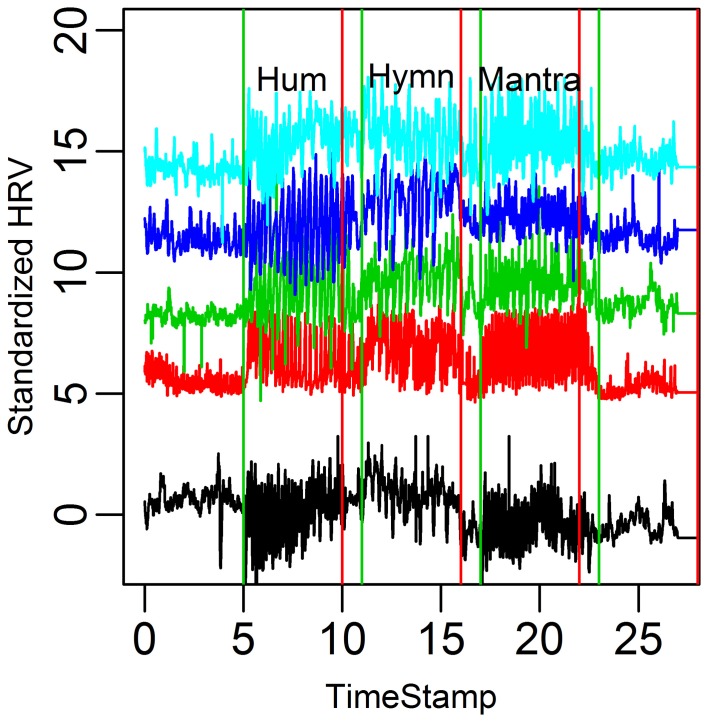
**HR graphs for the five subjects in the case study over the entire time domain**.

**Figure 10:**
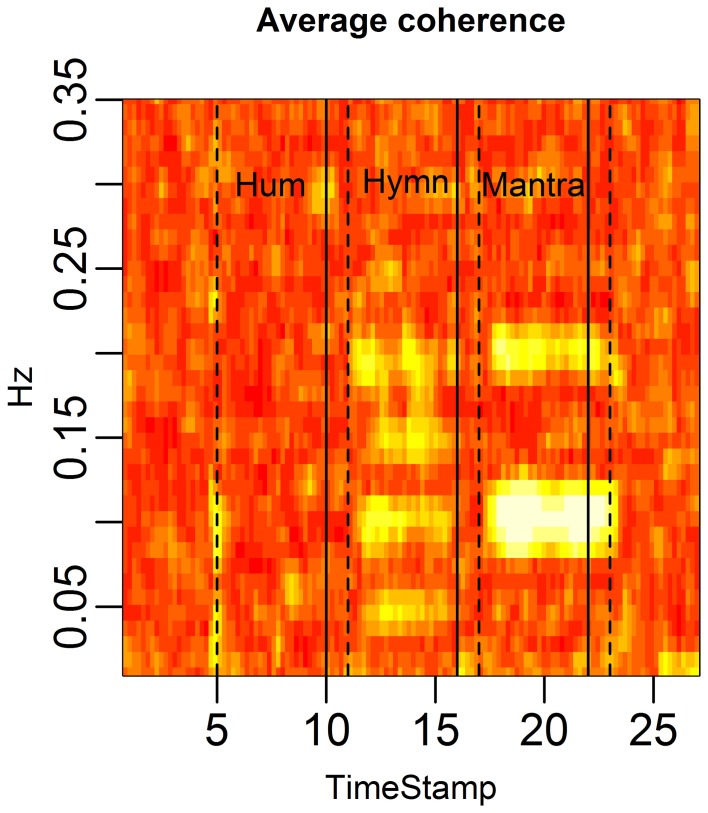
**HRV coherence for the case study.** Each column of the figure represents the average coherence across pairs of subjects for a certain time window. Each row represents a frequency in Hz. The coherence was computed in rolling windows of length 96 s, step size 12 s (cf. Figure 4). Coherence is clearly high during the mantra (at 0.1 Hz and at the harmonic frequency 0.2 Hz). There is also high coherence during the hymn (at 0.05, 0.1, and 0.2 Hz, and the harmonic 0.15 Hz).

**Figure 13:**
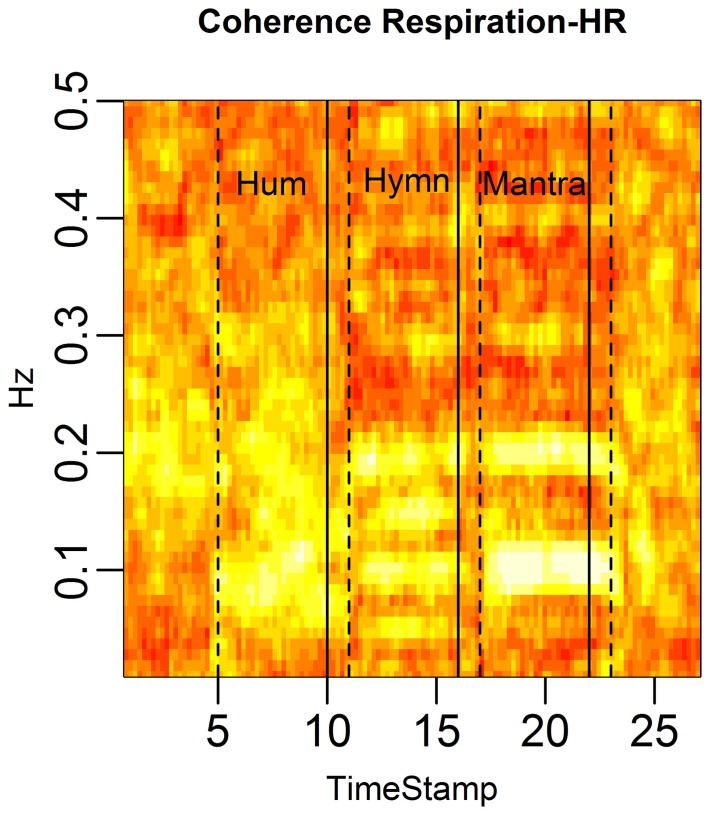
**RSA is defined as the coherence between respiration depth and HR.** We depict the average RSA across subjects in rolling windows of length 96 s, stepped by 12 s. Each column represents the coherence at different frequencies for a given time point and each row the coherence for a particular frequency across time. RSA is markedly high during the mantra (at 0.1 and the 0.2 Hz harmonic) as well as during the hymn (at 0.05, 0.1, and 0.2 Hz). RSA is also high during the hum segment, albeit not a common dominant frequency as expected since respiration frequency is highly individual during humming.

The correct order of the singing task labels is: “Hum” (5–10 min segment), “Hymn” (11–16 min segment) and finally “Mantra” (17–22 min segment).

Figures with correct labeling appear in this Erratum.

